# Quantitative Nuclear Magnetic Resonance Spectroscopy Based on PULCON Methodology: Application to Quantification of Invaluable Marine Toxin, Okadaic Acid

**DOI:** 10.3390/toxins8100294

**Published:** 2016-10-13

**Authors:** Ryuichi Watanabe, Chika Sugai, Taichi Yamazaki, Ryoji Matsushima, Hajime Uchida, Masahiro Matsumiya, Akiko Takatsu, Toshiyuki Suzuki

**Affiliations:** 1 National Research Institute of Fisheries Science, 2-12-4 Fukuura, Kanazawa, Yokohama 236-8648, Japan; rwatanabe@affrc.go.jp (R.W.); matsur@affrc.go.jp (R.M.); huchida@affrc.go.jp (H.U.); 2 Department of Marine Science and Resource, Nihon University, Kameino, Fujisawa 252-0880, Japan; world_wide_love_0511@yahoo.co.jp (C.S.); matsumiya@brs.nihon-u.ac.jp (M.M.); 3 National Institute of Advanced Industrial Science and Technology, Tsukuba Chuo 3, 1-1-1 Umezono, Tsukuba 305-8563, Japan; t-yamazaki@aist.go.jp (T.Y.); akiko-takatsu@aist.go.jp (A.T.)

**Keywords:** quantitative NMR, qNMR, PULCON, ERETIC2, external standard, okadaic acid

## Abstract

ERETIC2 (Electronic Reference To access In vivo Concentrations 2) based on PULCON (Pulse Length–based Concentration determination) methodology is a quantitative NMR (qNMR) using an external standard. The performance of the PULCON method was assessed using maleic acid (MA). Quantification of the diarrhetic shellfish toxin and okadaic acid by PULCON was successfully consistent with that obtained by a conventional internal standard method, demonstrating that the PULCON method is useful for the quantification of invaluable marine toxins without any contaminations by an internal standard.

## 1. Introduction

Diarrheic shellfish toxins (DSTs), okadaic acid (OA) [1] and its analogues, dinophysistoxins (DTXs) [2], are lipophilic marine toxins produced by toxic dinoflagellates *Prorocentrum lima* [3] and *Dinophysis* spp. [4]. DSTs may be accumulated in bivalves by filter-feeding the toxic dinoflagellates and can cause diarrhea and vomiting in humans when bivalves contaminated with DSTs are consumed [5]. Recently, the mouse bioassay, the traditional official testing method for DSTs in bivalves, has been replaced by LC/MS/MS [6] methodology in many countries, including Japan. Implementation of the instrumental methods requires certified reference materials (CRMs) of OA and DTXs with a defined concentration, purity and uncertainty.

NMR spectroscopy is a powerful analytical technique for structure elucidation of natural and synthetic organic compounds. Recently ^1^H-NMR has also been applied as a quantitative analytical technique as the NMR signal intensity is proportional to the number of nuclei under specific controlled conditions [7,8]. Quantitative NMR (qNMR) gives accurate and precise quantitative results for analytes when internal standards are used [9,10,11] since the concentration of analytes is directly determined via the integral value ratio. Thanks to the generic nature of ^1^H-NMR and qNMR as a quantitative tool, the technique has been used to determine purities of pharmaceutical products by assessing more than two separated signals on the products. However, qNMR is intrinsically useful for the quantification of analytes to prepare calibrant CRMs for instrumental analysis such as HPLC with UV, and fluorescence or mass spectrometric detection. The results obtained by the internal standard method are considered to be higher in accuracy and precision than those obtained by the external standard method. However, the use of internal standards results in the contamination of analytes which is a serious drawback in the quantification of compounds with difficulties in availability to prepare CRMs of precious marine toxins. Marine toxins, even though their molecules rarely exceed 1200 dalton in molecular mass, are complex and difficult-to-synthesize molecules. Hence, they are often obtained using a culture of dinoflagellates and painstaking isolation of the toxins which are secondary metabolites of these micro-organisms. The external standard method for quantification of the purified toxin has the advantage of recovering analytes without any contamination. Therefore, qNMR with external standards is more suitable to prepare CRMs for marine toxins [12].

ERETIC2 [13] (Electronic Reference to access in vivo Concentrations 2) is a commercial name by Bruker for the PULCON methodology (Pulse Length–based Concentration determination) [14], and it is a relatively new external standard technique in qNMR. Because PULCON correlates the absolute intensities in two one-dimensional (1D) NMR spectra by the principle of reciprocity [14], PULCON is easily applied to all NMR spectrometers. Despite the usefulness of the PULCON method, only a few studies on PULCON qNMR have been reported [15,16,17,18]. In our present study, the PULCON method was assessed by using the CRM maleic acid (MA) as a model compound and applied to the quantification (concentration determination) of OA to investigate our preparative scheme of CRM OA. By comparison with the internal standard method, the PULCON technique was assessed for its accuracy and its precision.

## 2. Results and Discussion

### 2.1. Performance Tests of PULCON Method with Different Analytical Conditions

The quantitation of MA dissolved in five different solvents, D_2_O, CD_3_OD, acetone-*d*_6_, DMSO-*d*_6_ and acetonitrile-*d*_3_, obtained by PULCON with a reference of MA (6.27 ppm) gave sufficient recoveries (Table 1). Recoveries obtained with protic solvents (D_2_O, CD_3_OD) were quantitative in comparison to those obtained by aprotic solvents, probably due to problems of solubility of MA rather than accuracy of the PULCON method. The average recovery in five solvents was 98.9% (1.5% RSD), suggesting that the PULCON technique allows for the quantification of analytes dissolved in different solvents. Frank et al. [15] also demonstrated that quantification of various compounds dissolved in a variety of solvents (acetonitrile-*d*_3_, methanol-*d*_4_, D_2_O, benzene-*d*_6_, D_2_O/DCl and DMSO-*d*_6_) using a caffeine/D_2_O solution as an external standard was in accordance with the results obtained by gravimetric method. Our results indicate that the PULCON technique enables the quantitation of compounds dissolved in various solvents, even when the external standard is dissolved in different solvents with samples.

**Figure 1:**
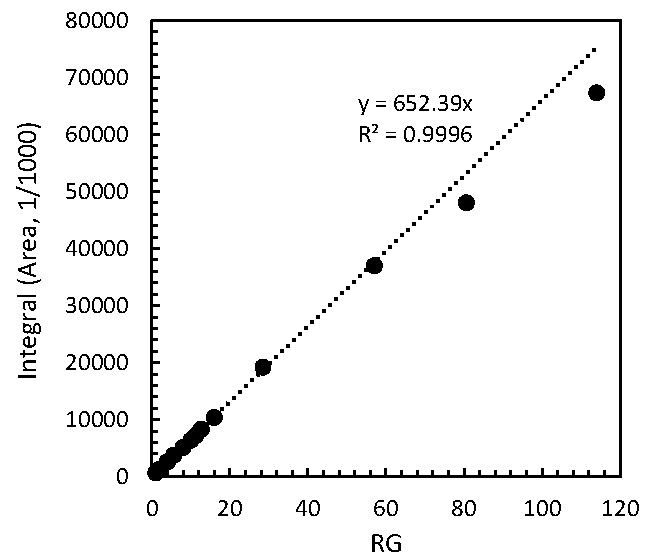
Relationship between receiver gain value and integral.

The effect of receiver gain on NMR quantitation was determined. The parameter is used to match the amplitude of the FID to the dynamic range of the digitizer [15]. The receiver gain (RG) values between the external standard and analyte are considered to be different due to different analytical conditions, including solvents or impurities of analytes. The MA reference (89.06 mM) was initially measured at a value of 6.35 on the RG which was set automatically by the *rga* command and then the analyte MA was measured at different RG values of 1, 6.35 and 10. The recoveries measured at RGs of 1 and 6.35 were 100.3% and were higher than that obtained at 10 (97.4%). The low recovery obtained at the RG value of 10 could be caused by the analogue-digital converter (ADC) overflow. Moreover, we investigated the linearity of the receiver using 3.03 mM MA/CD_3_OD solution. Figure 1 shows the relationship between the receiver gain and the integral. The integral area of MA was linear in the range from RG: 1 to RG: 60; however, beyond a RG of 60 the integral area was lower than expected due to ADC overflow, indicating that adequate values of RG must be used.

In the case of analytes at low concentrations, quantification of the analytes has to be done by increasing the number of scans. Therefore, the number of scans for the analyte would be different from that for the external standard. Thus, the effect of the number of scans on NMR quantitation was investigated (Table 2). NMR spectra of the reference solution (0.55 mM MA) were acquired over 64 scans, while the analyte in solution (3.69 mM MA/CD_3_OD) was measured at one, eight, 16, 32 and 64 scans, respectively. The recoveries in all experiments gave consistent results, despite largely differing numbers of scans (100.7% and 101.4%).

Table 3 shows the repeatability obtained for the analyte MA quantified by PULCON with a reference MA. The analyte was measured on three subsequent days and calculated as a recovery by using the PULCON method. Intra-day precision was 100.3% ± 0.3% (average ± S.D.), whereas inter-day precision was 99.8% ± 0.6%. The average recovery through three subsequent days was 99.8% ± 0.4%, demonstrating an excellent repeatability.

### 2.2. Relationship of MA Concentrations between Gravimetric and PULCON Method

**Figure 2:**
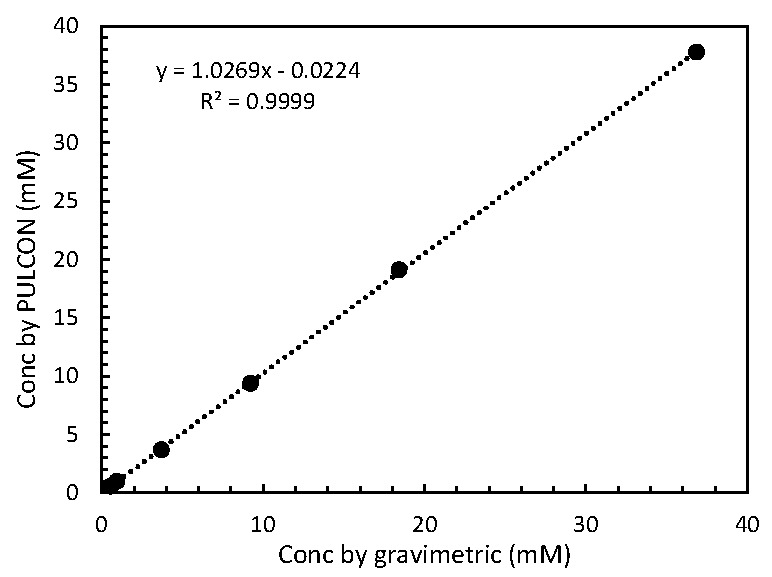
Linear regression of concentration of maleic acid determined by either gravimetric and PULCON methods.

MA/CD_3_OD solutions of 0.55, 0.92, 3.69, 9.22, 18.43 and 36.86 mM, respectively, were measured and quantified by the PULCON method using 0.55 mM MA/CD_3_OD solution as an external standard. The relationship between MA concentrations by gravimetric and PULCON methods was plotted (Figure 2). The linear regression coefficient was 0.999 and the slope was 1.02, indicating that PULCON gives good linearity and accuracy compared to the gravimetric method. Frank et al. [15] also showed that the PULCON method gave a good linearity compared to gravimetry in the quantification of benzoic acid, caffeine, and L-tyrosine. 

### 2.3. Comparison of OA Concentrations between PULCON Method and Internal Standard Method

**Figure 3:**
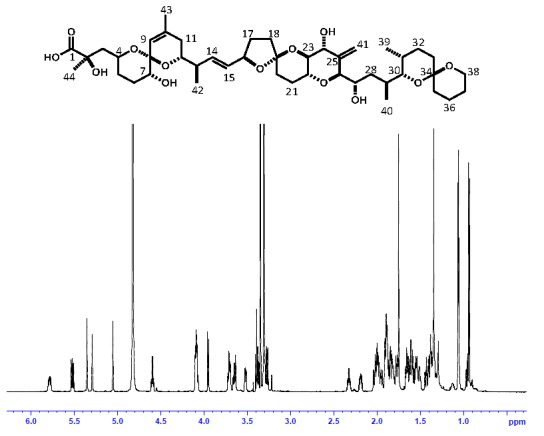
Chemical structure and ^1^H-NMR spectrum of okadaic acid dissolved in CD_3_OD (800 MHz).

OA was isolated from a large culture of the toxic dinoflagellate *Prorocentrum lima* by liquid-liquid partitioning and several column chromatography steps [19]. The purity analysis of OA using NMR demonstrated sufficient purities of more than 95%, with signals of impurities hardly being detectable in the range from 0 to 8 ppm (Figure 3). The five well-separated signals at 5.78, 5.52, 5.36, 5.30 and 5.06 ppm corresponding to H-14, H-15, H-41, H-9 and H-41 of OA, respectively, were used for quantitation. A slight fluctuation of the baseline was noticed in the spectra after baseline correction; this effect was detected in signal integration with both methods (Table 4). In addition, signal 1 gave lower concentration values compared to the other signals, which is possibly related to conformational disorder in the OA skeleton [20]. Therefore, signal 1 was excluded from calculations for quantitation. 

Generally, the internal standard method gives higher precision and accuracy than the external standard method. Therefore, the precision and accuracy of PULCON were compared with those of the internal standard method. Each method was performed in duplicate. In both methods, the concentrations obtained in those signals were averaged in each tube and then the average concentration in each tube was averaged. The OA concentration (average ± S.D.) was calculated by the internal standard with 1,4-BTMSB-*d*_4_ gave 352.8 ± 7.5 µg/g. On the other hand, the concentration quantified by PULCON gave 355.4 ± 0.1 µg/g, corresponding to 100.7% of the values obtained by the internal standard method (Table 4). OA is a lipophilic polyether compound with a medium molecular size of MW 804.5 in lipophilic marine toxins. Because quantification of OA by PULCON with 1,4-BTMSB-*d*_4_ external standard gave a relevant result, PULCON can be useful for several lipophilic polyether marine toxins with availability difficulties.

## 3. Conclusions 

We conducted performance tests of the PULCON method using CRM MA as a model compound. The PULCON method gave quantitative results for MA with an external standard prepared in various solvents (acetonitrile-*d*_3_, methanol-*d*_4_, D_2_O, acetone-*d*_6_, and DMSO-*d*_6_). It was noticed that adequate values of the receiver gain must be used to obtain quantitative results with the PULCON method. The PULCON method showed good linearity and repeatability in the quantification of MA. It was demonstrated that the PULCON method was also applicable to the quantification of OA as a diarrhetic shellfish toxin without any contaminations by an internal standard.

## 4. Materials and Methods 

### 4.1. Reagents and Chemicals

Maleic acid (TraceCERT grade, Lot: BCBG2002V, 99.99% ± 0.09%, *k* = 2) was purchased from Fluka (Tokyo, Japan). Traceable reference material of 1,4-bis (trimethylsilyl) benzene-*d*_4_ (1,4-BTMSB-*d*_4_, Lot:KPQ4815, 99.9% ± 0.5%, *k* = 2, TraceSure grade) were purchased from Wako pure chemicals (Tokyo, Japan). Deuterated solvents; D_2_O 99.96 atom%D (Aldrich, Tokyo, Japan), CD_3_OD 99.8 atom%D (Wako pure chemicals, Tokyo, Japan), CD_3_OD 100.0 atom%D (Acros organics, Yokohama, Japan), acetone-*d*_6_ 99.9 atom%D (Aldrich), DMSO-*d*_6_ 99.9 atom%D and acetonitrile-*d*_3_ 99.8 atom%D (Cambridge Isotope Laboratories, Tokyo, Japan) were used. NMR tubes used in the study were standard NMR tube with 5 mm i.d. (Kusano Science Corp., Tokyo, Japan). OA was purified and isolated by liquid-liquid partitioning and several column chromatography steps from a large culture of toxic dinoflagellate *Prorocentrum lima* [19].

### 4.2. NMR Instrument, Data Acquisition and Data Processing on Performance Tests

All NMR spectra were acquired by a Bruker AVANCE III spectrometer (^1^H: 800 MHz) with a cryogenic probe (CPTCI 5 mm ^1^H/^19^F-^13^C/^15^N/D Z-GRD) using standard NMR tubes at 298 K. The data acquisition and data processing were performed using the Topspin 3.0 software (Bruker, Kanagawa, Japan). Prior to the data acquisition, the 90° pulse length was calibrated. Sample was well-tuned and matched automatically. The acquisition parameters used for performance tests were set as follows; Pulse sequence: ‘zg’, relaxation delay (D1): 30 s, spectral width (SW): 20 ppm, data acquisition time (AQ): 3 s, flip angle (P1): 90° (ca. 9.0 µs), dummy scans (DS): 8, number of scans (NS): 64, DigMod: Baseopt, spinning: OFF. Relaxation delay time of 30 s was sufficient, which is more than 10 times T1 (1.47 s) of MA. Receiver gain (RG) was automatically set by Topspin 3.0 software (RG: 4). In particular, pulse calibration was performed as follows: 360° pulse was at first roughly calculated in 0.5 µs interval within 10 µs (e.g., 30–40 µs) and then the accurate 360° pulse was calculated in 0.03 µs interval within 1 µs. the other parameters; D1: 30 s, SW: 20 ppm, AQ: 2 s, DS: 0, NS: 1. The obtained NMR spectra were processed by multiplying with exponential (0.3 Hz line broadening) and zero-filling. The phases were corrected manually, and then the baseline was corrected by fifth-order polynomial. Peak integration was manually selected. The slope and bias corrections of the integral were not used. The concentration of analyte was quantified by using PULCON method in Topspin 3.0 software. Individual integral values, concentration and the number of protons in the signal used for quantitation in the reference were entered to PULCON software and then the concentration of signal in sample was automatically calculated.

### 4.3. Sample Preparation for Performance Tests of PULCON Method

MA of 31.75 mg was precisely weighed using electronic force balance XS205 (0.01 mg precision, Mettler Toledo, Tokyo, Japan), and then was mixed with 20 mL of methanol (5000-fold concentrated, Kanto chem., Tokyo, Japan) using a 20 mL volumetric flask (20 ± 0.03 mL at 20 °C, SHIBATA, Tokyo, Japan). Aliquots (20 µL) of the MA solutions were put into 2 mL glass vials by using gas-tight glass syringe and then dried up using nitrogen gas. The dried MA was dissolved in 500 µL deuterated solvents (CD_3_OD) using 0.5 mL gas-tight glass syringe and then transferred into NMR tubes. This solution of 0.547 mM was used as reference.

Subsequently, to assess the effect of different solvents on quantitation, MA solutions were prepared as follows: MA of 33.21 mg was precisely weighed using electronic force balance XS205 and then was dissolved in 20 mL of methanol using a 20 mL volumetric flask. Aliquots (100 µL) of the MA solution were divided in each 2 mL glass vial by using 100 µL gas-tight glass syringes, and then were dried up using nitrogen gas. The dried MAs were dissolved in 500 µL of deuterated solvents (D_2_O, CD_3_OD, acetone-*d*_6_, DMSO-*d*_6_ and acetonitrile-*d*_3_) using 0.5 mL gas-tight glass syringe and then transferred into NMR tubes.

Furthermore, MA solutions for performance tests were prepared as follows. MA of 42.81 mg was precisely weighed using electronic force balance XS205 and then was dissolved in 20 mL of methanol using a 20 mL volumetric flask. Aliquots (1000, 500, 250, 100, 25 µL) of the MA solution were divided in each 2 mL glass vial by using gas-tight glass syringes, and then were dried up using nitrogen gas. The dried MAs were dissolved in 500 µL of CD_3_OD using 0.5 mL gas-tight glass syringe and then transferred into NMR tubes.

### 4.4. Comparison of OA Concentrations between PULCON Method and Internal Standard Method

Sample preparation for internal standard method was performed as follows. One milliliter of OA solution (ca. 300 µg/mL) was weighed precisely using XS205 electronic force balance and a gas-tight syringe, and then dried up by nitrogen gas for over 72 h. The dried OA was dissolved in 1 mL of 1,4-BTMSB-*d*_4_/CD_3_OD solution at a final concentration of 1.000 mg/g, 0.8 mL of which was transferred into an NMR tube. The test was performed twice. The NMR spectra were acquired by an 800 MHz Avance III spectrometer on the following acquisition parameters. Pulse sequence: ‘zgig’, D1: 60 s, SW: 40 ppm, AQ: 6 s, P1: 90° (ca. 9.0 µs), DS: 16, NS: 512, ^13^C decoupling: ON (90 ppm offset), temperature: 298 K, spinning: OFF. T1 for signals 1 to 4 of OA was 0.62, 1.26, 0.57 and 1.15, and 0.67 s, respectively. The relaxation delay time of 60 s was sufficient because it is over 10 times T1 of OA signals. As the baseline obtained was fluctuated, the baseline was manually corrected using the *.baslpts* command. Peak integrals were manually selected. The slope and bias corrections of the integral were not used. The OA concentration was calculated from integral ratio of an internal standard signal vs. olefinic signals in OA. The concentration obtained of each signal was averaged in each tube and then the data repeatedly acquired were averaged.

Sample preparation for PULCON method was performed as follows. OA solution was prepared according to the internal standard method. On the other hand, 1,4-BTMSB-*d*_4_ solution as an external standard was prepared at a final concentration of 1.000 mg/mL CD_3_OD (6.4579 mM), 0.8 mL of which was transferred into an NMR tube. The test was performed twice. The basic data acquisition and data processing parameters were the same as the above internal standard method. As the baseline obtained was fluctuated, the baseline was manually corrected using the *.baslpts* command. However pulse sequence used was ‘zg’, and ^13^C decoupling was off to avoid sample heating as it was observed in internal standard method preceding performed. OA concentration was calculated from integral values, concentration and the number of protons in 1,4-BTMSB-*d*_4_ by using PULCON method in Topspin 3.0 software. The concentration obtained of each signal was averaged in each tube and then the data repeatedly acquired were averaged. In the experiments, 1,4-BTMSB-*d*_4_ was used as internal standards because MA was possible to overlap by being shifted the olefinic signals (especially signal 1) of OA depending on concentration [20].

## Figures and Tables

**Table 1:** Effect of different deuterated solvents on NMR quantitation ^1^.

Solvents	Recovery (%)	RSD (%, *n* = 3)
D_2_O	100.3	0.0
CD_3_OD	100.3	0.3
Acetone-*d*_6_	97.9	0.0
DMSO-*d*_6_	98.1	0.2
Acetonitrile-*d*_3_	97.1	0.2
Average	98.9	-
Stdev	1.5	-
RSD (%)	1.5	-

^1^ 0.55 mM maleic acid in CD_3_OD was used as reference. Sample was used 2.86 mM maleic acid (*n* = 3).

**Table 2:** Effect of number of scans on NMR quantitation ^1^.

Number of Scans	Recovery (%)
1	100.7
8	101.4
16	101.4
32	101.4
64	100.7
Average	101.1
Stdev	0.4
RSD (%)	0.4

^1^ 0.55 mM maleic acid in CD_3_OD was used as reference.

**Table 3:** Repeatability (%) of maleic acid with intra-day and inter-day precision ^1^.

Samples	Day 1	Day 2	Day 3	Average	Stdev	RSD (%)
1	99.7	100.3	99.5	99.8	0.4	0.4
2	99.5	100.5	99.5	99.8	0.6	0.6
3	99.7	100.0	99.2	99.6	0.4	0.4
Average	99.6	100.3	99.4	-	-	-
Stdev	0.2	0.3	0.2	-	-	-
RSD (%)	0.2	0.3	0.2	-	-	-

^1^ 0.55 mM maleic acid in CD_3_OD was used as reference. Sample was used 3.69 mM maleic acid in CD_3_OD.

**Table 4:** Quantitation results obtained by internal standard method and PULCON.

Method	Tube	Signal	Integral Region	Quantitation (µg/g)			Average	Stdev	RSD (%)
				Run1	Run2	Run3			
Internal standard method	1	1	5.85–5.72	344.30	344.08	340.38	342.92	2.20	0.64
		2	5.57–5.49	346.23	345.58	344.36	345.39	0.95	0.28
		3	5.38–5.25	345.44	349.27	354.62	349.78	4.61	1.32
		4	5.09–5.03	367.78	332.26	345.23	348.42	17.98	5.16
	2	1	5.85–5.72	346.39	344.29	349.36	346.68	2.55	0.74
		2	5.57–5.49	362.29	360.85	357.01	360.05	2.73	0.76
		3	5.38–5.25	365.55	361.48	355.51	360.85	5.05	1.40
		4	5.09–5.03	361.77	355.68	355.76	357.74	3.49	0.98
PULCON	1	1	5.86–5.72	344.81	333.91	340.59	339.77	5.50	1.62
		2	5.58–5.48	359.29	355.49	355.08	356.62	2.32	0.65
		3	5.41–5.24	363.20	360.73	338.95	354.29	13.34	3.77
		4	5.10–5.01	371.00	363.92	364.74	366.55	3.87	1.06
	2	1	5.86–5.72	339.47	351.09	342.76	344.44	5.99	1.74
		2	5.58–5.48	351.92	360.04	357.88	356.61	4.21	1.18
		3	5.41–5.24	353.67	359.63	342.35	351.88	8.78	2.49
		4	5.10–5.01	359.73	365.08	366.31	363.71	3.50	0.96
